# Anti-Photoaging and Potential Skin Health Benefits of Seaweeds

**DOI:** 10.3390/md19030172

**Published:** 2021-03-22

**Authors:** Ratih Pangestuti, Kyung-Hoon Shin, Se-Kwon Kim

**Affiliations:** 1 Director of Research and Development Division for Marine Bio Industry, Indonesian Institute of Sciences (LIPI), West Nusa Tenggara 83352, Indonesia; ratih.pangestuti@lipi.go.id; 2 Department. of Marine Science and Convergence Engineering, College of Science and Technology, Hanyang University, Gyeonggi-do 11558, Korea; shinkh@hanyang.ac.kr

**Keywords:** photoaging, seaweeds, skin, health, bioactive

## Abstract

The skin health benefits of seaweeds have been known since time immemorial. They are known as potential renewable sources of bioactive metabolites that have unique structural and functional features compared to their terrestrial counterparts. In addition, to the consciousness of green, eco-friendly, and natural skincare and cosmetics products, their extracts and bioactive compounds such as fucoidan, laminarin, carrageenan, fucoxanthin, and mycosporine like amino acids (MAAs) have proven useful in the skincare and cosmetic industries. These bioactive compounds have shown potential anti-photoaging properties. Furthermore, some of these bioactive compounds have been clinically tested and currently available in the market. In this contribution, the recent studies on anti-photoaging properties of extracts and bioactive compounds derived from seaweeds were described and discussed.

## 1. Introduction

The skin is the largest organ of the integumentary system and one of the most complicated organs in the body. It has many function such as covering the internal organs, maintaining body temperature, preventing water loss, and acting as a protective physical barrier from the external (environment) stimulus, damaging pathogens, pollutions and ultraviolet (UV) radiation [1]. Though UV radiation (UVR) has many beneficial effects, the skin’s prolonged exposure to UVR could be an aggressive factor for photoaging and mutations which cause cancer [2]. 

Melanocytes produce melanin as part of the skin’s self-photoprotection, and possess biological properties (i.e., radical scavenging) [3,4]. The UVR directly and or indirectly induced the activation of complex signaling cascade in human skin [5]. This process begins by absorbing electromagnetic energy through cellular chromophores and then converting it into chemical energy. Furthermore, these energized chromophores react and produce generation of reactive oxygen species (ROS) which further leads to the activation of a wide range of transcription factors in skin cells such as activator protein-1 (AP-1) and nuclear factor kappa B (NF-κB) [6,7]. The AP-1 induces the upregulation of matrix metalloproteinases (MMP) such as collagenase-1 (MMP-1), stromelysin-1 (MMP-3), and gelatinase A (MMP-2), which specifically degrade connective tissues such as collagen and elastin and indirectly inhibit the collagen synthesis in the skin [8]. Furthermore, prolonged UVR exposure is considered as a leading cause of photoaging, and its symptoms could be characterized by wrinkles, loss of skin tone, pigmentation (hypo- or hyperpigmentation), rough skin, dryness, sallowness, deep furrows, severe atrophy, melanoma, and many others [9,10,11]. Therefore, it is important to provide adequate photoprotection to prevent photoaging and other skin disorders due to the deleterious effects of UVR. 

Various synthetic or semi synthetic materials have been used as anti-photoaging agents. However, these materials have limited use due to their instability and adverse side effects such as potential toxicity and potency to interfere with certain pathways of the multistage process of carcinogenesis [12]. In addition, consumers are conscious and demand more natural, green, and eco-friendly products with beneficial claims for the skin [13]. The increased demand of natural anti-photoaging products has led to a number of research works and innovation on nature-derived anti-photoaging agents. 

**Figure 1:**
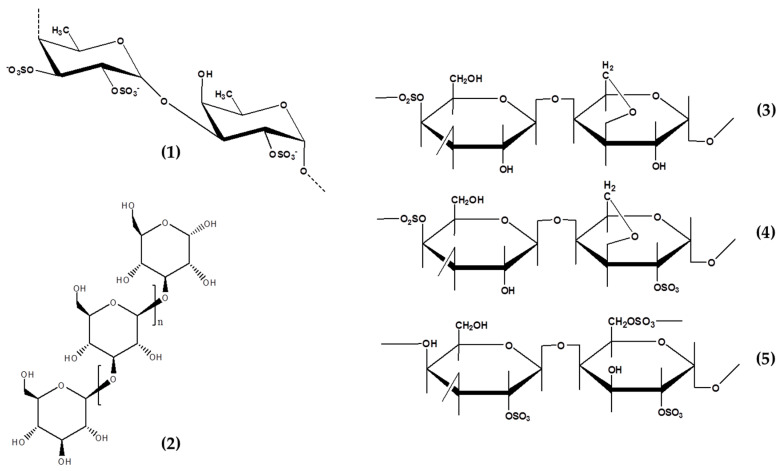
Chemical structure of seaweeds derived polysaccharides showed potential anti-photoaging properties. Fucoidan, **1**; Laminarin, **2**; Kappa-carrageenan, **3**; iota-carrageenan, **4**; and lambda-carrageenan, **5**.

Bioactive compounds from marine plants including seaweeds have proven to be a great source of novel materials for incorporating into anti-photoaging formulations [14,15]. The number, extraction, purification, and characterization of anti-photoaging compounds from marine sources are high and competitive compared to other marine floras such as sea grass, mangroves, and marine faunas (i.e., teripang or sea cucumber, sea star, sea urchin, and more). Furthermore, seaweed polysaccharides including fucoidan, laminarin, and carrageenan (Figure 1) showed potential anti-photoaging properties which were mediated by intra-cellular ROS scavenging activity in UV irradiated cells and in vivo models [16,17,18,19]. Other seaweed-derived materials such as mycosporine like amino acids (MAAs) are well known as the most potential natural UVA-absorbing molecules [13]. Moreover, their extracts are also continuously reported as potential anti-photoaging agents [20]. 

**Figure 2:**
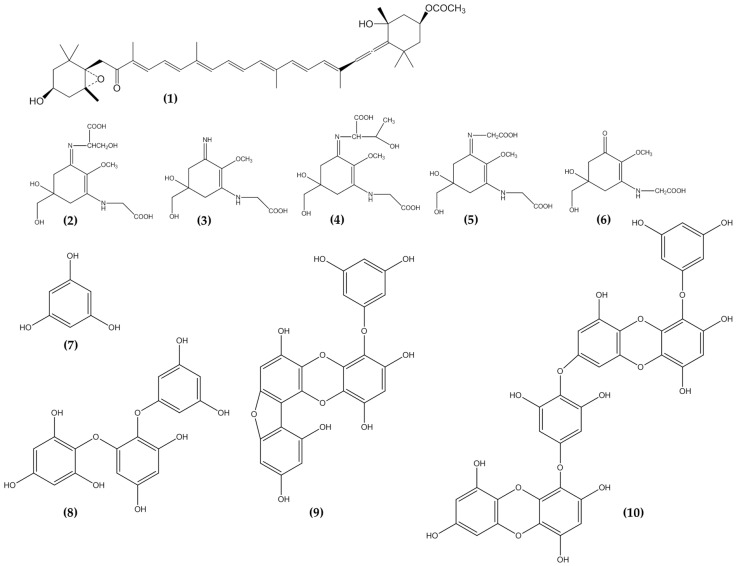
Carotenoids, Mycosporine like amino acids and Phlorotannins from seaweeds showed potential anti-photoaging properties. Fucoxanthin, **1**; Shinorine, **2**; Palythine, **3;** Porphyra-334, **4**; Asterina-330, **5**; Mycosporine-glycine, **6**; Phloroglucinol, **7**; Triphlorethol-A, **8**; Fucofuroeckol-A, **9**; and Dieckol, **10**.

Therefore, bioactive compounds from seaweeds (Figure 1 and Figure 2) have attracted great interest and are known for promoting distinct functional activities of interest for the development of anti-photoaging products. They exhibit great ability in targeting several key players linked to anti-photoaging properties such as radical scavenging activity, strong UV absorption, inhibit cell death, MMP-1, and other activities [14,21]. This review focused on the anti-photoaging potential of seaweed-derived bioactive compounds and extracts. The most relevant and up to date studies on anti-photoaging agents found in seaweeds and their biological roles were further summarized and discussed. In addition, the potential role they play in skincare and cosmetic products were also elaborated. 

## 2. Seaweeds Extracts as Potential Anti-Photoaging Agents

Seaweeds are autotrophic organisms that are widely distributed and consist of a wide variety of species. Based on the pigment contents they can be classified into red, green, and brown seaweeds [22]. Furthermore, they are found in intertidal shores to a depth of 150 m and are highly exposed and susceptible to UVR. Therefore, to counteract and minimize photodamage induced by high UVR, seaweeds synthesize photoprotective materials. 

The anti-photoaging activities of seaweed extracts have been demonstrated in various in vitro and in vivo models (Table 1). The activities were mainly mediated by antioxidant properties, radical scavenging activity, and UV absorbing capacity. Furthermore, many seaweed extracts, especially red types possess significant levels of anti-photoaging activities. However, little attention has been paid towards the anti-photoaging properties of green seaweed extracts. The aqueous extract of *Halimeda incrassata* and *Caulerpa* sp showed anti-photoaging activity in UVC-irradiated plasmid DNA and UVB irradiated mice, respectively [23,24]. Furthermore, more than 20 seaweed species belonging to Rhodophyceae and Phaeophyceae obtained from several countries including Spain, Chile, Ireland, South Africa, Argentina, and Tonga were tested [25]. Compared to others, three species belonging to Rhodophyceae; *Macrocystis pyrifera*, *Porphyra columbina, Sarcothalia radula* and *Gigartina skottsbergii* exhibited the highest photoprotective activity. The authors correlated this photoproetective activity with total phenolic contents (TPCs). However, it might also correlate with high MAA contents in Rhodophyceae. For example, *Porphyra yezoensis* (also known as laver) extract showed strong photoprotective activity on the UVB-irradiated human keratinocytes (HaCaT) cells. While laver extract showed absorbance spectrum characteristics of MAAs in red algae and contained high phenolic compounds [26], it also showed the absorbance spectrum characteristic of major MAAs including porphyra-334 or shinorine. 

Recently, the anti-photoaging properties of two Antarctic red seaweeds, *Curdiea racovitzae* and *Iridaea cordata*, have been described [27]. Both red seaweeds extract contains high concentrations of MAAs (palythine, asterina-330, and shinorine). Meanwhile, the total MAAs content of *C. racovitzae* and *I. cordata* are 150.17 and 60.78 (μg MAAs/mg extract), respectively. Furthermore, when compared with *I. cordata, C. racovitzaei* showed better photoprotective properties which might be correlated with higher MAAs contents. It also showed better ROS scavenging activity than quercetin and Helioguard 365^®^ (anti-aging product containing MAAs Porphyra-334 and Shinorine; Mibelle Biochemistry, Switzerland). These red Antarctic seaweeds showed great potential for being developed as anti-photoaging agents. 

The UV filters could be used in skincare and cosmetic products to protect the skin from harmful effects of UVR. Currently, many commercial UV filter products not only contain synthetic or semi synthetic UV filters, but they are also complemented with extracts and bioactive compounds from natural resources. In addition, products complemented with natural anti-photoaging agents are more effective in overcoming the undesirable effects of UVR. For example, the combination of *Porphyra umbilicalis* extracts, vitamins and *Ginkgo biloba* were able to improve the photoprotective performance of sunscreens, thereby preventing UVR-induced photodamage [28]. Therefore, seaweed extract could be added to anti-photoaging and sunscreen formulations in order to prevent oxidative stress and improve the absorption spectra of UV filters.

## 3. Seaweed Compounds as Potential Sources of Anti-Photoaging Agents

Seaweeds are heavily loaded with potential reservoirs of bioactive compounds such as polysaccharides, MAAs, natural pigments, phenolic compounds, proteins, peptides, and others. Previous studies have also investigated the anti-photoaging properties of bioactive compounds from seaweeds. In addition, further research has been carried out on the most studied seaweed-derived bioactive compounds and extracts as potential anti-photoaging agents.

### 3.1. Polysaccharides Rich Extract 

The photoprotective activity of polysaccharides rich extract from brown seaweeds (*Hizikia fusiforme, Sargassum fusiforme, Sargassum vachellianum,* and *Ecklonia maxima*) was investigated and monosaccharide analysis showed that most of its rich extract contains sulfate group and a high amount of fucose (43.20 to 53.53 %) (Table 2). In addition, it was found that fucose-containing sulfated polysaccharides possessed various bioactivities and most of the polysaccharides rich extract were able to inhibit ROS production and down regulated MMP expression (especially MMP-1) [21,45,46,47,48,49]. This suggests that the anti-photoaging activity of polysaccharides rich extract from brown seaweeds was mainly mediated through antioxidant and MMP inhibitory activity. 

The anti-photoaging properties of two fucoidan-rich seaweed extracts from *Undaria pinnatifida* and *Fucus vesiculosus* have been demonstrated. Both brown seaweeds extracts showed inhibitory activity against enzymes related to skin aging process. Clinical testing showed that both extracts were able to protect skin from UVR and wrinkle depth reduction. In addition, *F. vesiculosus* extract which contain polysaccharides and high polyphenol demonstrated additional efficacy in antioxidant and skin brightening benefits [50]. In addition, the mixture of fucose and rhamnose in skincare formulation has been claimed to inhibit skin ageing process [51]. 

Polysaccharides rich extracts from brown seaweeds are potentially developed as anti-photoaging agents in skincare or cosmetic products. Furthermore, when added in skincare or cosmetic products formulations, they improved the efficacy and maintained the skin in good condition especially due to their moisturizing properties. It is believed that some polysaccharides might also improve the stability and sensorial properties of cosmetic and skincare products.

### 3.2. Fucoidans

Fucoidans, sulfated polysaccharides, have been isolated from different brown seaweeds species. These compounds have attracted great interest in the food and cosmetic industries [16]. Furthermore, there are many studies that focused on the isolation, characterization, and medicinal values of fucoidans and the anti-photoaging properties of fucoidan. 

The antioxidant activity of fucoidan has been determined by several radical scavenging methods and the most common are 1,1-diphenyl-2-picryl hydrazil (DPPH), superoxide anion, and hydroxyl radical scavenging assays. Fucoidan have exhibited both primary (chain-breaking antioxidants) and secondary (radical scavengers) antioxidants. The primary antioxidant potential of fucoidan is characterized by its ability to react directly with free radicals and convert them to more stable non-radical products [52,53,54]. Furthermore, the strong secondary antioxidant potential of fucoidan extracted from *Sargassum binderi*, *Sargassum* spp, and *Undaria pinnatifida* has been reported [55,56,57]. Its antioxidant activities are strongly related with sulfate content and molecular weight (MW). However, low molecular weight (LMW) fucoidan has shown more antioxidant potentials compared to synthetics antioxidant (Butylated hydroxyanisole; BHA) and higher MW fucoidan. Koh et al. (2019) suggested that the sulfate groups in the LMW fucoidan are more accessible compared to the ones with high molecular weight (HMW), thereby resulting in remarkably higher secondary antioxidant activity. 

**Figure 3:**
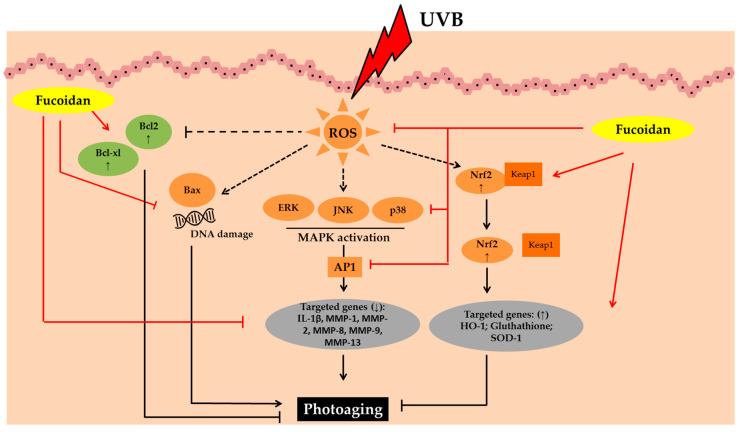
Photoprotective activity of fucoidan. Abbreviations: B-cell lymphoma-2 (Bcl-2), B-cell lymphoma-extra-large (Bcl-xL), Bcl-2-associated *X* protein (Bax), Reactive oxygen species (ROS), Mitogen-activated protein kinases (MAPK), c-JUN N-terminal kinase (JNK), Extracellular signal-regulated kinase (ERK); Activator protein 1 (AP1), interleukin-1β (IL-1β), Matrix metalloproteinase (MMP); Nuclear factor erythroid 2–related factor 2 (Nrf2), Kelch Like ECH Associated Protein 1 (Keap1), Heme oxygenase-1 (HO-1);Superoxide dismutase 1 (SOD-1); Down-regulated, decreased (↓); up-regulated, increased (↑).

The photoprotective activity of fucoidan has been studied using UVB irradiated HaCaT and human foreskin fibroblast (HS 68) cells, zebrafish, and in vivo models [19,58,59,60,61,62,63,64,65,66,67]. The earliest study on the photoprotective activity of fucoidan was carried out by Kim et al. in 2008. They demonstrated its photoprotective activity in UVB-irradiated HS 68 cells via MMP-1 inhibition and ERK pathways [58,60]. Furthermore, the photoprotective activity of fucoidan from *Hizikia fusiforme* was observed in UVB-induced photodamage in human dermal fibroblasts (HDF) cells and zebrafish models. Its treatment significantly inhibited collagenase and decreased the intracellular ROS levels. Furthermore, it significantly inhibited intracellular collagenase, reduced the expression of MMP, and improved collagen synthesis in UVB-irradiated HDF [68]. The summary of the potential photoprotective activity of fucoidan is shown in Figure 3. Fucoidan is extensively explored for its photoprotective properties and being isolated from several brown seaweed species such as *Costaria costata, Fucus evanescens, Sargassum hemiphyllum, Sargassum horneri, Sargassum siliquastrum, Ecklonia cava, Saccharina japonica*, and *Hizikia fusiforme*. The biological activities are affected by many factors such as seaweed species, MW, purity, sugar composition, sulfation degree, co-extracted impurities, glycosidic linkage, and branching site [69]. In addition, it was found that the bioecology and harvesting months/seasons also influenced the composition and biological activities [70]. Mak et al. (2013) studied the monthly variations of fucoidan content in *U. pinnatifida* and it was found that the sporophyll part of *U. pinnatifida* consistently contained the highest amount compared to the frond part. Furthermore, it was found that the sporophyll maturation of *U. pinnatifida* strongly affected the fucoidan content and composition [70]. 

Generally, this polysaccharides contains sulfate, fucose as the main sugar, uronic acids, acetyl groups, protein, and other monosaccharides (such as mannose, glucose, galactose, xylose, and rhamnose) [71,72]. The structures and monosaccharide compositions of fucoidans from different brown algae sources vary from different species. Recently, Ponce et al., (2020) provided a comprehensive study on the compositional data of fucoidans from different brown seaweeds species. Its monosaccharide composition is strongly related with taxonomic classification and an example includes polysaccharides extracted from the genus Fucus which are classified as being rich in fucose (>70% of monosaccharides). Meanwhile, in order Laminariales, the presence of sulfated galactofucans with high galactose content is almost equal to the fucose content [72]. The composition and sulfation degree of fucoidan is strongly affected by extraction and purification methods. Therefore, there is a need to develop suitable extraction techniques to maintain its composition and sulfation pattern in order to obtain the desired bioactivity [73,74]. 

Lower molecular weight has been reported to enhanced the biological activity of fucoidan [16]. Therefore, in order to obtain LMW and stronger bioactivities; chemical, radical, acidic, and enzymatic hydrolysis are generally used. Previous studies have shown that treatment with LMW fucoidan shows stronger photoprotective activity than HMW [62,63,64]. Hwang et al. (2017) provided detailed a extraction process and characterization of photoprotective activity of HMW, LMW, desulfated, and acetylated fucoidan isolated from *S. hemyphyllum* (Table 3) [62]. Furthermore, LMW fucoidan showed stronger protection against UVB-induced HS 68 cells. These results suggest that its fucose content, sulfation, and MW play an important role in photoprotective activity. Supporting these results, Kim et al. (2018) stated that LMW fucoidan treatment inhibits photoaging by enhance antioxidant and anti-inflammatory activities and inhibiting extracellular matrix degradation in UVB-irradiated HR-1 (hairless) mice. It is mostly absorbed prior to UVB irradiation. Therefore, it is assumed that LMW fucoidan may involve in photoprotective effects rather than UV filtering. The LMW fucoidan extracted from *S. horneri* showed a stronger photoprotective activity compared to HMW in UVB-irradiated HaCaT cells [64]. 

Pozharitskaya et al. (2019) investigated the pharmacokinetics of fucoidan after topical application in rats. It was found that ointment contains 15% fucoidan are distributed into the skin, striated muscle, and plasma with area under concentration-time curve for topical dose (AUC)_0–48_ = 0.94 µg·h/g, 2.22 µg·h/g, and 1.92 µg·h/mL, respectively [75]. The longest half-life for fucoidan was observed in plasma, striated muscle and skin. In addition, its accumulation in plasma was not observed after repeated topical applications of 100 mg/kg for five days. Collectively, it may be assumed that topical treatment with cream containing fucoidan have efficacy and safety benefits with little concern of accumulation and toxicity. In addition, these results suggest the potential of fucoidan as an anti-photoaging agent in skincare and cosmetic industries.

### 3.3. Carrageenans

Carrageenans are natural polysaccharides extracted mainly from red seaweeds (i.e., *Eucheuma* spp, *Chondrus crispus* (Irish moss), and *Gigartina stellate*). They are joined by α-1, 3 and β-1,4 glycosidic linkage by alternate units of d-galactose and 3,6-anhydrogalactose [76]. Twenty percent of carrageenan production are used in pharmacy, skin care, and cosmetics products, and this is due to their unique physical functional properties (i.e., thickening, gelling, emulsifying, and stabilizing properties) [77]. The tree main types of commercially available carrageenan include kappa (κ; forms strong, rigid gels in the presence of potassium ions), iota (ι; forms soft, clear, and elastic gels in the presence of calcium ions) and lambda (λ; does not form gel and normally used to thicken dairy products) [17]. 

In addition to their thickening and gelling properties, carrageenans have also shown potential antioxidant activities. De Souza et al. (2007) tested the antioxidant activity of κ, ι and λ carrageenan and based on radical scavenging assay, λ carrageenan had better results [78,79]. Furthermore, it was found that the degradation into carrageenan oligosaccharides enhanced its antioxidant activity [80]. Previous studies have shown that polysaccharides with LMW had stronger antioxidant activity compared to HMW polysaccharides. These activities may be related to the ability of LMW polysaccharides to have more reductive hydroxyl group terminals which further affect the ability to accept and eliminate free radicals. In addition Sun et al. (2015) reported that the antioxidant activities of carrageenan oligosaccharides could be related to the sulfate group, the degree of polymerization, the reduction of sugar, and the structure of reducing terminus [81].

Thevanayagam et. al. (2013) stated that the photoprotective effects of κ-, ι- and λ-carrageenan in UVB-irradiated HaCaT cells [82]. All carrageenan types tested in their study showed significant protection against detrimental effects of UVB-induced apoptosis in HaCaT cells and scavenge free radicals. In addition, many studies have investigated the antioxidant activities of carrageenans [80,81,83,84]. 

In addition, the anti-photoaging activity of carrageenan also correlates with the modulations of inflammatory responses. These polysaccharides are able to induce the activation of proinflammatory mediators such as of tumor necrosis factor (TNF)-α, interleukin (IL)-6, IL-1β, inducible nitric oxyde synthase (iNOS), and cyclooxygenase-2 (COX-2) [85]. Furthermore, Tripp et al. (2003) found that COX-2 expression is an important factor for keratinocyte survival and proliferation after acute UV irradiation. The inhibition of COX-2 expression has been demonstrated to reduce epidermal keratinocytes proliferation [86]. Therefore, it is believed that the modulation of inflammatory responses and antioxidant activities of carrageenan may play an important role in their anti-photoaging activity. Purwaningsih et al. (2015) formulated a sunscreen cream with carrageenan and black mangrove fruit (*Rhizopora mucronata*). It was found that a sunscreen formula containing 0.5% carrageenan and 1% *R. mucronata* extract showed high photoprotective properties compared to other formulas tested in their study [87]. 

The photoprotective activities of carrageenan reported in previous study might reflect its new potential in skin care and cosmetic industries rather than just being used as an excipient. There are numerous advantages of these polysaccharides over other bioactive substances, including relatively low production costs, safety, non-toxic properties, wide acceptability, suggesting carrageenan as a promising anti-photoaging candidate in the near future; however, further studies such as formulations in order to obtain the most optimum anti-photoaging properties are required.

### 3.4. Laminarins

Laminarins are storage polysaccharides extracted from brown seaweeds and composed of (1–3)-β-d-glucan with β-(1–6) branching with different reducing endings either mannitol or glucose residues. Laminarin has been extracted from several brown algae species such as *Eisenia bicyclis, Saccharina longicruris, Laminaria digitata, Laminaria hyperborean, Laminaria japonica, Sargassum mcclurei, Cystoseira barbata*, and *Durvillaea potatorum* [88,89,90,91]. 

In vivo studies have shown the anti-photoaging potential of laminarin and an example is the study conducted by Li and colleagues (2013) which was based on the effect of laminarin on the activity of MMP-1 of photoaging skin in mice models. The laminarin treatment significantly increased the thickness of dermis, tissue inhibitor MMP-1 (TIMP-1) level, and decreased the expression and release of MMP-1 [92]. It also protected mouse dorsal skin from UVB induced photodamage [93]. Furthermore, it significantly increased collagen fibers in the dermis of the UVB treated ICR mice. Laminarin pretreatment provided photoprotection by decreasing oxidative stress and increasing antioxidant enzymes including superoxide dismutase (SOD)-1, SOD-2, glutathione peroxidase (GPx), and catalase (CAT). In addition, it also showed photoprotective properties in UVA-irradiated HDF, HaCaT and normal human epidermal keratinocytes (NHEK) cells [89]. Treatment with laminarin attenuates pro-inflammatory cytokines (IL-6) levels and basal ROS levels in HDF and NHEK cells at concentration of 1 and 10 µg/mL. 

Many studies have reported the enhanced antioxidant activity of LMW laminarin [18,90,94,95,96,97,98]. This encouraged Choi et al. (2011) to prepare LMW laminarin by gamma irradiation and the formation of carbonyl groups by gamma irradiation was observed. Carbonyl groups were mainly attributed to the enhanced antioxidative activity of laminarin [95,96]. However, Rajauria et al. (2021) found that the purification of laminarin which involve solvents and molecular weight cut-off (MWCO) filters reduced the antioxidant activity compared to the crude laminarin extract [94]. In addition, chemical modifications (i.e., sulfation, carboxymethylation, acetylation, phosphorylation, and benzoylation) have affected the antioxidant activity of polysaccharides to some extent. The chemical modifications of laminarin via carboxylation using dielectric barrier discharge, conjugation with gallic acid, and sulfation have also been reported. Analyses of the chemical composition of carboxylated laminarin (LMC), gallic acid-conjugated laminarin (LMG), and sulphated laminarin (LMS) yielded 11.7% carboxyl groups, 1.5% gallic acid, and 1.4% sulfate content, respectively. This chemically modified laminarin was tested against several antioxidant assays including total antioxidant, hydroxyl radical scavenging, superoxide radical scavenging, iron chelating, reducing power and copper chelating assays. It was reported that LMG showed better antioxidant activities compared to other chemically modified laminarin [98].

Interestingly, Sellimi et al. (2018) showed that the topical application of laminarin-based creams improved the wound healing process in rats by accelerating the collagen deposition and re-epithelization and protected the skin cells from oxidative stress [91]. It appears to be a promising skincare and cosmetic ingredients for anti-photoaging agents. However, treatments with laminarin at high concentration have decreased the metabolic activity in dermal fibroblasts and keratinocytes cells [89]. Therefore, in order to be applied in skincare and cosmetics, further study on laminarin solubility, efficacy evaluation, penetration capacity, half-life time in blood, and bioavailability of laminarin needs to be carried out.

### 3.5. Phlorotannins

Polyphenolic compounds are a class of secondary metabolites which are categorized into several classes according to the number of phenol rings and structural elements that bind them together [43]. Phlorotannins are class of polyphenol compounds found exclusively in brown seaweeds and synthesized via acetate–malonate pathway (also known as the polyketide pathway) [99]. Furthermore, they are also known as seaweeds-chemical defense agents. These bioactive compounds protect seaweeds against grazers, important components of seaweeds cell wall and are responsible for the absorption of UVR [100]. 

Phlorotannins have been extracted from different brown seaweed resources such as Ecklonia cava, Ecklonia stolonifera, Sargassum thunbergii, Hizikia fusiforme, Endarachne binghamiae, Laminaria sp., and Sargassum piluliferum (Table 4). Out of the total brown seaweed species, E. cava was found to contain more total phenol contents [101]. Compared to other phlorotannins isolated from E. cava, phlorogucinol showed stronger cytoprotective effects in UVB-irradiated HaCaT cells. Currently, the anti-photoaging properties of phloroglucinol are far more explored compared to other phlorotannins. Phloroglucinol showed strong antioxidant activities by inhibiting hydroxyl radical, superoxide radical, and intracellular ROS, and induced the expression of antioxidant enzymes by activating the nuclear factor erythroid 2 (NFE2)-related factor 2 (Nrf2)/ heme oxygenase-1 (HO-1) signaling. Milanovic and colleagues (2020) studied the antioxidant activity of phloroglucinol and 2,4,6-Trihydroxypyridine towards HO· radicals. The study showed that phloroglucinol is a more powerful antioxidant compared to 2,4,6-Trihydroxypyridine [102]. Furthermore, it was found that the electron-withdrawing effect of nitrogen was stronger than the electron donating effect of the OH groups in the molecule of 2,4,6-Trihydroxypyridine. The structure difference of 2,4,6-Trihydroxypyridine with phloroglucinol is the substitution of nitrogen atom in the aromatic ring of phloroglucinol. Therefore, chemical modifications may affect the scavenging capacity of phloroglucinol. In addition, many studies have showed that the anti-photoaging activity of phlorotannins is strongly related to their radical scavenging activity. The hydroxyl (–OH) group bound to the aromatic ring donates electron and give it to a free radical or other reactive species. This underlies the inhibition of ROS and ROS-mediated damage on macromolecules, which in turn contributes to inhibiting the activation of the signal transduction pathways such as the NF-κB, mitogen-activated protein kinase (MAPK) signaling pathway.

Phlorotannins represent great potency as active anti-photoaging substances by providing multiple actions such as antioxidant, anti-inflammatory, MMP-inhibition, and down-regulation of pro-apoptotic factors. Based on to a certain level of concentration, they do not exert any toxic effect, anticipating its potential use as safe anti-photoaging agents in skin care and cosmetic products. The other biological activity such as anti-microbial activity of phlorotannins shows potency of phlorotannins as natural preservatives in skincare and cosmetic products. Therefore, besides functioning as anti-photoaging agents, they also show great potential to be used as skincare and cosmetic agents with other potential skin benefit effects. 

### 3.6. Mycosporine Like Amino Acids

Mycosporine-like amino acids are LMW, water-soluble molecules that strongly absorb UVA and UVB; generally MW of MAAs are (<400 ~Da) [120]. These colorless LMW molecules are widely distributed in natures and could be found in many organisms such as phytoplankton, terrestrial lichens, cyanobacteria, coral, cnidarians, sponges, shrimp, sea urchins, starfish, clams, ascidians, and seaweeds [121]. Differing with photosynthetic pigments, MAAs is invoked to function as a passive shielding substances by dissipating the absorbed radiation energy in the form of harmless heat without generating photochemical reactions [122]. Their absorption maxima are around 310 to 360 nm depending on the molecular structure [13,123]. Based on the structural view, MAAs consists of cyclohexenimine ring conjugated with two amino acid, amino alcohol or amino group substituents [124]. 

Mycosporine-like amino acids are demonstrated as one of the strongest naturally occurring UVA-absorbing molecules [13]. Currently, they have been identified from more than 500 seaweed species [125,126]. Furthermore, when compared to other seaweed classes, the red category is an excellent source of MAAs. Sun et al. (2020) stated that in seaweeds, they are mainly distributed in orders Bangiales, Ceramiales, Gigartinales, and Gracilariales. In Table 5, information is provided on several MAAs present in seaweeds. Furthermore, during the last five years, a growing number of papers focusing on anti-photoaging properties of MAAs from seaweeds have been observed. 

The anti-photoaging activities of MAAs are not only mediated by their photoprotective activity by absorbing UVR, but also by strong antioxidant, radical scavenging, macromolecule damage-protection, anti-inflammatory, MMP inhibitor, and other potential anti-photoaging activities. Furthermore, the antioxidant activity of seaweeds derived MAAs such as porphyra-334, shinorine, asterina-330, palythine and mycosporine -glycine (Myc-Gly) have been tested in various assays. These include 2,2′-Azinobis-(3-Ethylbenzothiazoline-6-Sulfonic Acid Assay (ABTS+) radical scavenging, β-carotene/ linoleate bleaching method, scavenging capacity of superoxide radicals, Oxygen Radical Absorbance Capacity (ORAC-fluorescein) Assay, ROS scavenging [127,132,133]. In general, MAAs showed strong antioxidant activities. However, the exact mechanisms are still unknown and further investigations need to be carried out on the antioxidant mechanisms of MAAs.

In addition, MAAs derived from seaweed also showed photoprotective activity in HaCaT cells by protecting DNA damage from UVB radiation [134]. Recently, it was demonstrated that Porphyra-334 and shinorine treatment activated Nrf2/Kelch-like ECH-associated protein 1 (Keap1) pathway. Porphyra-334 and shinorine first dissociated Nrf2 from Keap1. Increased mRNA expression of Nrf2 targeted genes encoding oxidative stress defense proteins prior and post UVR exposure were observed [135]. Treatment of shinorine and Porphyra-334 in UV irradiated mice was found to increase the expression of endogenous antioxidant (SOD, GSH-Px, CAT), and decrease malondialdehyde expression [136]. Seaweed-derived MAAs showed antioxidant properties through several functions which include strong UV absorption, protecting macromolecules damage, and antioxidant capacity.

Seaweeds-derived MAAs have also been tested for their anti-inflammatory properties in UV-irradiated HaCaT cells [137]. Porphyra-334 treatments suppressed COX-2 expression and one of the main cytotoxic mediators participating in the innate response in mammals [138]. In addition, Shinorine and Porphyra-334 treatment in LPS-stimulated macrophages cells showed potential anti-inflammatory properties. While MAAs treatment significantly suppressed the release of pro-inflammatory mediators which were mediated through NF-κB signaling pathway [139]. Supporting these results, Poprhyra-334 treatment in UV-irradiated mice also inhibited the activation of NF-κB and MAPK signaling pathways [140]. Furthermore, many intracellular signaling pathways are involved in inflammatory responses. However, NF-κB and MAPK are amongst the most important signaling molecules involved in inflammatory responses [141]. Collectively, these reports have showed MAAs as potential anti-inflammatory agents stimulated by UV-irradiation.

Collagen is the major structural protein of the extracellular matrix (ECM) that provides supportive framework to the cell and is responsible for strength, elasticity, and hydration of the skin. [142] Therefore, collagen and ECM play an important role in skin health, beauty, and aging. Porphyra-334 showed potential anti-photoaging properties by inhibiting MMP-1 and MMP-3 levels. Treatment of Porphyra-334 in human dermal fibroblast cells increase ECM components, such as procollagen, type I collagen, elastin [132,143]. Porphyra-334 also showed an inhibition of advanced glycation end products (AGEs) [143]. The results indicated that treatment with Porphyra-334 maintains the structural integrity of collagen fibers by absorbing ultraviolet radiation. Therefore, Porphyra-334 showed great potential function in preventing skin photoaging.

Among other seaweed-derived MAAs, Porphyra-334 is the most studied MAAs. They have also been reported to down regulate caspase-3 protein expression in UV irradiated HaCaT, suggesting another anti-photoaging properties were also mediated by the down-regulation of pro-apoptotic factors [134]. Suh et al. (2017) studied the expression profiling of Porphyra-334 modulated genes or microRNA (miRNAs) in response to UV-exposure and their functional networks. It was found that Porphyra-334 regulated Wnt (Wingless/integrase-1; related to UV-repressed genes) and Notch signaling pathways. Furthermore, it is assumed that Porphyra-334 protects cells from UV-induced photoaging through the comprehensive modulation of expression patterns of genes involved in UV-mediated biological processes [144].

Sunscreen cream containing 0.005% MAAs extracted from *P. umbilicalis* (nori) was found to neutralize photodamage caused by UVA radiation as efficiently as cream containing 1% synthetic UVA and 4% UVB filters [145]. Furthermore, the formulation of Porphyra-334 increased the photoprotective activity of sunscreen formula [146]. MAAs protects the skin cells by their ability to disperse harmful UV into heat that dissipates into the surroundings without forming reactive photoproducts. The treatment with MAAs was able to inhibit skin wrinkle depth, roughness, and elasticity. This suggests that MAAs are effective and potential anti-photoaging agents. In a recent article, it was found that sunscreen formulated with MAAs showed the same Sun Protecting Factor (SPF) and UVB-Biological Effective Protection Factors (BEPFs) as reference sunscreens but slightly lower UVA-BEPFs [147].

### 3.7. Carotenoids

Carotenoids are essential natural pigments along with chlorophylls in photosynthetic organisms, bacteria, and fungi. Furthermore, these tetraterpene pigments are involved in photosynthesis and photoprotection. Carotenoids can be classified into two broad groups, namely carotenes (contain no oxygen) and xanthophylls (oxygenated derivatives of carotenes) [148]. In 2018, around 850 carotenoids were been found, and the number is still increasing [149]. Among carotenoids isolated from seaweeds, fucoxanthin is a major xanthophyll with diverse biological functions. These carotenoids represent more than 10% of total carotenoids. 

**Figure 4:**
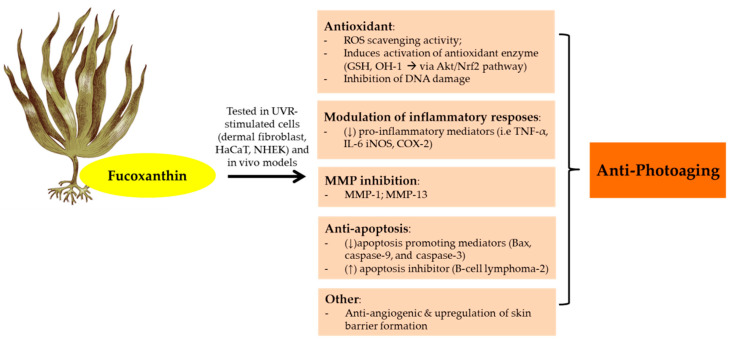
Anti-photoaging activity of fucoxanthin extracted from brown seaweeds. Abbreviations: Reactive oxygen speies (ROS); Human keratinocytes (HaCaT); Normal human epidermal keratinocytes (NHEK);Glutathione (GSH); Protein kinases B (Akt);Nuclear factor erythroid 2–related factor 2 (Nrf2); Matrix metalloproteinase (MMP); Tumor necrosis factor (TNF-α); Interleukin (IL); Inducible nitric oxyde synthase (iNOS); Cyclooxygenase-2 (COX-2); Down-regulated, decreased (↓); Up-regulated, increased (↑).

The anti-photoaging function of fucoxanthin has been investigated by many studies (Figure 4). As a consequence of UVB irradiation, cells face an intense oxidative reaction that gives rise to photodamage and photoaging. Furthermore, fucoxanthin isolated from Korean brown seaweeds *Sargassum siliquastrum* showed photoprotective properties in UVB-irradiated human fibroblast. A 24 h pretreatment with fucoxanthin (50–250 μM) were able to reduce oxidative stress via ROS scavenging activity and counteract UVB-induced cell damage in dose-dependent manner [150]. Furthermore, fucoxanthin also showed remarkable ROS scavenging activity in UVB-irradiated mice and HaCaT and HDF cells [151,152,153]. It showed strong antioxidant activity due to its singlet oxygen quenching (^1^O_2_) and ROS scavenging effects. From the structural view, fucoxanthin has a unique unusual allenic bond and 5,6-monoepoxide in its molecule which plays an important role in ROS scavenging activity [22]. In addition, functional groups in the terminal ring of fucoxanthin also have an effect in their antioxidant activity. The electron-rich status of fucoxanthin makes this carotenoid an effective radical scavenger [154]. 

Inflammatory stimuli could trigger MMP which leads to photoaging, and when UVB reaches our body, keratinocytes which represent the first target act as sentinels, initiate the signaling cascade. These events address the stress and the production of pro-inflammatory factors such as NO, PGE_2_, IL-6, IL-1β and TNF-α. Furthermore, Luna et al., (2018) showed that pretreatment of HaCaT cells with fucoxanthin at 50 µM reduced the downstream inflammatory cytokines (TNF-α and IL-6) [155]. In addition, the synergy effects of fucoxanthin and rosmarinic acid (phenolic ester isolated from *Rosmarinus officinalis* L) on UVB-exposed HaCaT have been demonstrated [156]. A combination of fucoxanthin (5 µM) and rosmarinic acid (5 µM) improved the antioxidant and anti-inflammatory profiles compared to individual compounds. The photo-protective effects of fucoxanthin and rosmarinic acid were mediated by down-regulation of NLR family pyrin domain containing 3 (NRLP3)-inflammasome and upregulation of Nrf2 signaling pathway which further increased the antioxidant gene expression (HO-1).

The levels of structural proteins for the epidermal permeability barrier, including filaggrin (filament aggregating protein) markedly decline in aged skin. UVR has been associated with the level of filaggrin, based on in vitro and in vivo experimental models [157]. Following UV exposure, filaggrin gene expression was down regulated. Furthermore, treatment with 0.5% fucoxanthin (4 days until day 8) stimulates filaggrin promoter activity and upregulates filaggrin gene expression [158]. This upregulation of the skin barrier formation by fucoxanthin may contribute to the photoprotective porperties of fucoxanthin. In addition, its treatment protects HaCaT cells from hydrogen peroxide-induced cell death. Fucoxanthin protective actions were mediated by the down-regulation of apoptosis promoting mediators (Bcl-2-associated *X* protein (Bax), caspase-9, and caspase-3) and the up-regulation of apoptosis inhibitor (B-cell lymphoma-2 Bcl-2) [152]. 

Continuous exposure to UV irradiation induces skin angiogenesis and wrinkle formation [159]. Furthermore, the topical administration of fucoxanthin (0.001%) prior to UVB radiation in hairless mice showed potential anti-angiogenic effects. It also diminishes epidermal hypertrophy, MMP-13 expression in the epidermis and thiobarbituric acid reactive substances (TBARS) in the skin [151]. Other studies also showed that fucoxanthin treatment ameliorated UVB irradiation-induced corneal damage and down-regulating Vascular endothelial growth factor (VEGF) expression [160]. 

The possibility of administering fucoxanthin topically faces several drawbacks because of the issue of lipophilicity and HMW. Anti-photoaging agents need to diffuse across the stratum corneum and tight junctions to achieve effective permeation. Several vehicle such as hydrogel, cream, and ointment have been tested to achieve the best permeation results with cream showed the most favorable vehicle for fucoxanthin topical administration [155]. Furthermore, a cream containing fucoxanthin was applied in UVB-irradiated erythema model in hairless mice. It showed photoprotective properties through the down-regulation of COX-2 and iNOS and the up-regulation of HO-1 protein via Nrf-2 pathway. In addition, the effects of fucoxanthin (0.5% in alkyl benzoate or in EtOH) in reconstructed human skin have also been investigated and it was found that its topical applications were safe. Fucoxanthin treatment upon UVB irradiation in reconstructed human skin ameliorated pro-inflammatory mediators (IL-6 and IL-8) [161]. Collectively, it is believed that fucoxanthin could be a natural adjuvant for preventing photoaging.

## 4. Potential of Seaweeds in Anti-photoaging Products 

### 4.1. Seaweed Diversity Opens Untapped Potential for Anti-Photoaging Products

Currently, more than 30,000 species of algae have been reported with about 15,000 species belonging to macroalgae (including terrestrial and seaweeds) [162]. These huge numbers of algal diversity offer great potential to be applied in the food, pharmacy, cosmetic and skincare industries. However, many seaweed species are still considered to be underexploited resources. An example is in Indonesia, where 1000 seaweed species have been reported. These marine organisms play an important ecologic and socioeconomic role in coastal communities and drive economic growth. However, only a few species have been commercialized (i.e., Kappaphycus alvarezii; previously known as Eucheuma cottonii, Eucheuma spinosum and Gracilaria sp) [15]. All commercialized seaweeds are commonly used in the hydrocolloid industry to produce agar and carrageenan. Hundreds of seaweed species are still categorized as under-explored, and these conditions also happen in many countries. The anti-photoaging properties of many seaweeds species remain unexplored and these renewable marine resources have an untapped potential to be developed in skincare and cosmetics industries. 

### 4.2. Development of Sustainable Aquaculture to Support Seaweeds Potential in Skincare and Cosmetic Industries 

Twenty three percent of the world’s aquaculture production is from seaweeds, however, ‘marine agronomy’ is still in its infancy and seaweed potentials are still categorized as under-exploited (far from being fully exploited) [163]. With more seaweeds being used because of their anti-photoaging and other potential skin benefit effects, the demand for the use of seaweeds in cosmetic and skincare industries will grow globally. Therefore, the development of sustainable production of seaweed species through aquaculture is required [164]. To enhance seaweed production, it is important to understand and modify the main parameters that affect their cultivation. The parameters include water current and movement, water temperature, irradiance and photoperiod, nutrients dispersion and water quality, and the relationships of these factors and the intrinsic physiological responses [165].

The development of seaweed co-culture with other marine commodities can be easily carried out. Co-culture of seaweed with other marine flora or fauna could be carried out through a system called Integrated Multi-Trophic Aquaculture (IMTA). The IMTA system provide advantages environmentally, particularly in sustainability aquaculture, and social economic aspect [166]. Furthermore, it has also been useful in desired bioactive compound optimizations. 

Seaweed’s reproduction and the synthesis of anti-photoaging compounds may occur after some stress stimulus. For example, IMTA of *Rhodymenia pseudopalmata* with commercial marine fish (common snook; *Centropomus undecimalis*) under exposure to high solar radiation has also been investigated. While that of *R. pseudopalmata* increased anti-photoaging compounds such as MAAs (Porphyra-334, shinorine, palythine, palythinol, palythene usujirene, and asterina-330), radical scavenging activities, phenol, and natural pigment contents [167]. The culture of *Gracilaria vermiculophylla* in outdoor tanks in seawater with the addition of fishpond effluents in an IMTA system has also been demonstrated [168]. The highest MAAs content was observed in April and four MAAs were identified (Porphyra-334, Shinorine, Palythine and Asterina-330). Furthermore, the IMTA development of seaweed with other marine commodities could be used to optimize anti-photoaging compounds and provide another economic benefit for seaweed farmers.

### 4.3. Sustainable and Environmentally Friendly Extraction

Multiple solvent extractions have been used with other common methods to obtain anti-photoaging compounds from seaweeds. The extraction process involves a combination of various solvent such as methanol, n-hexane, dichloromethane, chloroform, and acetone. However, most of the solvent used in the extraction process are of safety concerns due to the hazardous, toxicity and impact to the environment. Many research groups have developed sustainable extraction technologies to obtain anti-photoaging compounds from seaweeds. These includes microwave assisted extraction (MAE), ultrasound assisted extraction (UAE), supercritical fluid extraction (SFE) and pressurized liquid extraction (PLE) [169]. These green technologies were able to extract anti-photoaging compounds from seaweed effectively. 

Many studies have extracted one of the anti-photoaging compounds (fucoxanthin) from *U. pinnatifida* with various methods from solvent extraction to SFE, MAE and UAE (Table 6). *U. pinnatifida*, is one of the most studied brown seaweed species by many research groups in Japan, Korea, and China. Based on the extraction yield of fucoxanthin, SFE may be considered as the best non-conventional extraction technique. One of the advantages of this system is the use of CO_2_ as a solvent, an easy-available compound which is non-toxic [170]. The SFE systems could be an efficient and respectful option for the production of fucoxanthin for cosmetic and skincare industries. However, these extraction processes have some weakness which includes cost of the installations and special manpower to operate it. In addition to extraction processes, sample pre-treatment could be another factor used in optimizing the extraction yield of anti-photoaging compounds from seaweeds.

Through the use of an environmentally friendly technology PLE, Saravana et al. (2018) optimized the extraction of fucoidan from *Saccharina japonica*. These processes involve solvent pressurized at certain temperature and pressure under critical level conditions (subcritical region). Under this condition, seaweed–solvent matrix is treated to a pressurized temperature from 100 to 374 °C with a pressure that is up to 22 MPa, and the solvent is maintained in the liquid state by operating with a constant pressure higher than that of the vapor. Furthermore, the subcritical condition facilitates an increase in dielectric constant and decrease in density which cause hydrocarbons to become more soluble, allowing complex reactions like decomposition and depolymerization to occur [171]. It was found that the most optimal conditions in PLE process for fucoidan extraction were extraction at 127.01 °C, 80 bar, S/L ratio of 0.04 g/mL, agitation speed of 300 rpm for 11.98 min [172]. The PLE process produced a high yield of fucoidan (13.56%) with good functional activity. Therefore, PLE might be the favored method for the extraction of fucoidan in the skincare and cosmetic industries. 

Collectively, many anti-photoaging substances could be extracted using environmentally friendly technologies. Selecting an extraction process is a key factor in achieving optimum extraction yields, desired biological activity and reduce production costs. In addition, the use of environmentally friendly extraction may increase the commercial value of the final cosmetic or skincare product. This is because there is usually a high demand for more natural, non-toxic, and ecofriendly products.

### 4.4. Potential of Seaweeds-Derived Anti-Photoaging Products in the Market 

The skincare and cosmetic industries have become two of the fastest growing and prosperous industry sectors [178]. The demand for new and innovative anti-photoaging products are continuously growing. Seaweeds anti-photoaging products have been developed and are commercially available in the market. *Undaria pinnatifida* extracts (containing 85% fucoidan) and *Fucus vesiculosus* extracts (containing 60% fucoidan and 30% polyphenol) have been tested in clinical studies. Both seaweed extracts increased the expression of sirtuin 1 (SIRT1), a protein known for its longevity-boosting and anti-ageing activity. Furthermore, clinical testing established the efficacy of the extracts in a range of tested applications, relative to placebo. The anti-photoaging properties of *U. pinnatifida* extract is modulation of skin immunity, soothing and protection, while *Fucus vesiculosus* extract significantly affected age spot reduction and increased brightness, soothing, and protection [50]. These brown seaweed extracts are currently available in the market under Marinova’s (Biotech Company from Autralia). In addition, other anti-photoaging extracts containing MAAs are also available on the market (Table 7). Seaweed derived anti-photoaging compounds need to be explored and are highly recommended as an active ingredient in sunscreen, anti-photoaging cream, moisturizers in skincare and cosmetics. In addition, environmentally friendly technology need be developed so the eco-friendly cosmetic and skincare could be more available in the market. 

## 5. Conclusions

There are still large opportunities to explore seaweed-derived bioactive compounds as anti-photoaging agents in the skincare and cosmetic industries. Furthermore, more studies need to be carried out on the sustainable culture of seaweeds and their optimization in order to obtain optimal bioactive compounds. There are still challenges involving the use of environmentally friendly technology for industrial applications. Therefore, the development of seaweeds in skincare and cosmetic industries is important but poses a challenge for scientists, engineers, seaweed farmers, skincare and cosmetic formulators, and product developers.

## Figures and Tables

**Table 1:** Anti-photoaging and mechanisms of various seaweed extracts.

Class	Species	Origin	Extracts	Test	Functions	Mechanisms	Ref
Rhodophyceae	*Solieria chordalis*	France	MeOH extract/CPC fractionation *n*-heptane/EtOAc//MeOH/dW (19/1//19/1; *v*/*v*)	-	Photoprotective	UV absorption	[29]
					Antioxidant	DPPH radical scavenging activity	
	*Bryothamnion triquetrum*	Cuba	Aqueous extract	UVC-irradiated plasmid DNA	Photoprotective	(↓) DNA dmage	[23]
	*Porphyra umbilicalis*	France	Cosmetic formula (5% extract) with Ginkgo biloba, vitamins	UVA/B-irradiated mice	Cell renewal	transepidermal water loss (TEWL) and erythema index.	[28]
					Anti-apoptosis	(↓) p53 and caspase-3	
	*Porphyra yezoensis*	Korea	EtOH extract (80%)/Chl/MeOH/dW (2/1/0.9)	UVB irradiated HaCaT	Photoprotective	Absorb UVB rays	[26]
					Antioxidant	(↑) GSH/GSSG ratio	
	*Gelidium amansii*	Korea	Mix with *Cirsium japonicum*; MeOH extract and fermentation	UVB-irradiated HS 68 DF& SKH-1 hairless mice	Inhibit collagen degradation; wrinkle formation	(↑) type I pro-collagen; (↓) MMP-1; -2; -9; -13	[30]
	*Polyopes affinis*	Korea	EtOH extract	UVB irradiated HaCaT	Antioxidant	(↓) intracellular ROS; (↓) superoxide radical (↓) hydroxyl radical; (↓) cellular damage	[31]
					Anti-apoptosis	NA	
					Photoprotective	Absorb UVB rays	
	*Solieria chordalis*	France	EtOAc; 2-OD and OE L-PCA extract	-	Photoprotective	Absorb UVB rays	[32]
						Protect synthetic chlorophyll sol. from UVB	
	*Polysiphonia morrowii*	Korea	80% EtOH	UVB irradiated HaCaT	Antioxidant	(↓) intracellular ROS; (↑) antioxidant enzyme	[33]
					Anti-apoptosis	(↓) TUNEL-positive cells and DNA fragmentation	
	*Chondracanthus tenellus*	Korea	80% EtOH	UVB irradiated HaCaT	Antioxidant	(↓) intracellular ROS; (↓) superoxide radical (↓) hydroxyl radical; (↓) cellular damage	[34]
					Anti-apoptosis	NA	
					Photoprotective	Absorb UVB rays	
	*Bonnemaisonia hamifera*	Korea	80% EtOH	UVB irradiated HaCaT	Antioxidant	(↓) intracellular ROS; (↓) superoxide radical (↓) hydroxyl radical	[35]
					Anti-apoptosis	(↓) TUNEL-positive cells and DNA fragmentation	
					Photoprotective	Absorb UVB rays	
	*Lomentaria hakodatensis*	Korea	80% EtOH	UVB irradiated HaCaT	Antioxidant	(↓) superoxide radical; (↓) hydroxyl radical	[36]
					Anti-apoptosis	(↓) DNA fragmentation (↓) apoptotic bodies	
					Photoprotective	Absorb UV rays	
	*Macrocystis pyrifera*	Argentina	Ace extract	UVB irradiated zebrafish embryo	Photoprotective	Survival of normal embryos (100%)	[25]
	*Porphyra columbina*	Argentina	Ace extract			Survival of normal embryos (100%)	
	*Sarcothalia radula*	Spain	Ace extract			Survival of normal embryos (91.7%)	
	*Gigartina skottsbergii*	Argentina	Ace extract			Survival of normal embryos (73.6%)	
	*Curdiea racovitzae*	Antarctic	MeOH, aqueous extract	UVA irradiated fibroblast	Photoprotective	Absorb UVA and UVB rays	[27]
						(↑) cell proliferations	
					Antioxidant	(↓) DPPH radical; ROS;(↓) superoxide radical	
	*Iridaea cordata*	Antarctic	MeOH, aqueous extract	UVA irradiated fibroblast	Photoprotective	Absorb UVA and UVB rays	[27]
						(↑) cell proliferations	
					Antioxidant	(↓) DPPH radical; ROS; (↓) superoxide radical	
Chlorophyceae	*Halimeda incrassata*	Cuba	Aqueous extract	UVC-irradiated plasmid DNA	Photoprotective	(↓) DNA damage	[23]
	*Caulerpa* sp.	Indonesia	EtOH extract	UVB irradiated mice	Inhibit collagen degradation	(↓) MMP-1;	[24]
Phaeophyceae	*Sargassum muticum*	Korea	80% EtOH; EtOAc fraction	UVB irradiated HaCaT	Antioxidant	(↓) intracellular ROS; (↑) antioxidant enzyme	[37,38,39]
					Anti-apoptosis	(↓) TUNEL-positive cells and DNA fragmentation; regulation of MAPK- and caspase-dependent signaling pathways; (↑) Bcl-2 and Mcl-1; (↓) Bax; (↓) caspase-9 and caspase-3	
					Photoprotective	Absorb UVB rays	
					Inhibit collagen degradation	(↓) MMP-1; (↓) AP-1	
	*Sargassum glaucescens*	Taiwan	Aqueous extract	UVA irradiated HaCaT	Antioxidant	(↓) intracellular ROS; (↑) antioxidant enzyme	[40]
	*Sargassum cristafolium*	Indonesia	EtOH extract	UVA irradiated HeLa; BALBL/c mice	Photoprotective	Absorb UVA rays; (↓) cellular damage	[41]
	*Fucus spiralis*	Portugal	EtOH;Cyclohex; EtOAc; Et2O; aqueous extract;	UVB irradiated HaCaT	Photoprotective	Absorb UVA; UVB; UVC rays	[20]
					Antioxidant	(↓) intracellular ROS; (↑) antioxidant enzyme	
	*Mazzaella laminarioides*	Chile	Ace extract	UVB irradiated zebrafish embryo		Survival of normal embryos (91.7%)	[25]
	*Undaria crenata*	Korea	80% EtOH	UVB irradiated HaCaT	Antioxidant	(↓) intracellular ROS; (↓) superoxide radical (↓) hydroxyl radical;	[42]
					Anti-apoptosis	(↓) apoptotic bodies and DNA fragmentation	
					Photoprotective	Absorb UVB rays	
	*Carpomitra costata*	Korea	80% EtOH	UVB irradiated HaCaT	Antioxidant	(↓) intracellular ROS; (↓) superoxide radical (↓) hydroxyl radical; (↑) antioxidant enzyme	[43]
					Anti-apoptosis	(↑) Bcl-2; (↓) Bax(↓) caspase-9 and caspase-3	
	*Ecklonia stolonifera*	Korea	80% EtOH	UVA irradiated HDF	Antioxidant	(↓) intracellular ROS;	[44]
					Inhibit collagen degradation	(↓) MMP-1; -3	

Abbreviations: Ethanol (EtOH); Methanol (MeOH); Ethyl aetate (EtOAc); Diethyl ether (Et_2_O); Cyclohexane (Cyhex); Centrifugal partition chromatography (CPC); Distilled water (dw); Chloroform (Chl); Acetone (Ace); 2-octyldodecanol (2-OD); Octyldodecyl ester of l-pyrrolidone carboxylic acid (OE L-PA); Ultraviolet (UV); Human keratinocytes (HaCaT); Human foreskin fibroblast (HS 68); Human Dermal Fibroblast (HDF); Human cervical cancer cells (HeLa); Reactive oxygen species (ROS); Glutathione (GSH); Oxidized glutathione (GSSG); Transepidermal water loss (TEWL); Activator protein 1 (AP1); Matrix metalloproteinase (MMP); Terminal deoxynucleotidyl transferase dUTP nick end labeling (TUNEL); Bcl-2-associated *X* protein (Bax); Nuclear factor erythroid 2–related factor 2 (Nrf2); Heme oxygenase-1 (HO-1); not available (NA); Down-regulated, decreased (**↓**); Up-regulated, increased (**↑**).

**Table 2:** Composition of seaweed rich polysaccharides extract showing anti-photoaging activity.

Algae Source	*Hizikia fusiforme*	*Sargassum fusiforme*	*Sargassum vachellianum*	*Ecklonia maxima*
Carbohydrate (%)	NA	58.10	53.51	69.37
Sulfated polysaccharide (%)	63.56	NA	NA	NA
Sulfated group (%)	NA	13.18	12.32	10.51
Xylose (%)	17.37	5.90	3.5	NA
Galactose (%)	23.15	18.40	9.3	NA
Glucose (%)	NA	1.50	2.20	NA
Fucose (%)	53.53	43.20	49.5	NA
Rhamnose (%)	NA	3.50	NA	NA
Fructose (%)	NA	18.50	NA	NA
Mannose (%)	NA	9	11.2	NA
Glucuronic acid (%)	NA	15.35	1.01	NA
	[46,47]	[48]	[21]	[49]

**Table 3:** Sulfate, fucose, and average molecular weight of fucoidan showing photoprotective activity.

Algae Source	*S. hemiphyllum*	*S. hemiphyllum*	*E. cava*	*S. horneri*
Fucose	208.2 ± 2.3 (μmol/g)	210.9 ± 3.3 (μmol/g)	NA	37.43%
Sulfate (%)	40.1 ± 0.9	38.9 ± 0.4	NA	28.01 ± 0.50%
Average MW (kDa)	270	0.8	~8	60
Ref	[62]	[62]	[63]	[64]

Abbreviations: Molecular Weight (MW); Kilodalton (kDa).

**Table 4:** Phlorotannin extracted from brown seaweed with potential anti-photoaging activity.

Phlorotannins	Seaweeds	Origin	Anti-Photoaging	Ref
Eckol	*Ecklonia stolonifera; Ecklonia cava*	Korea	Inhibit NF-κB, AP-1, MMP-1 expression Protect UVB-induced cell damage; (↓) Pro-inflammatory mediators	[101,103,104]
Dieckol	*Ecklonia stolonifera*	Korea	Inhibit NF-κB, AP-1, MMP-1 expression Protect UVB-induced cell damage; (↓) Pro-inflammatory mediators	[103,104,105,106]
Phloroglucinol	*Ecklonia cava*	Korea	(↓) hydroxyl and superoxide radical, intracellular ROS; (↑) SOD, GSH; Activate Nrf2/HO-1 Inhibit NF-κB, MAPK; MMP-1 expression (↓) Bax; Caspase-3 (↓) Pro-inflammatory mediators	[101,107,108,109,110,111]
Triphlorethol-A	*Ecklonia cava*	Korea	Protect UVB-induced cell damage; (↓) intracellular ROS; Inhibit MAPK; MMP-1 expression (↓) Caspase-3 and -9 Strong absorption in UVB spectra	[101,112,113]
Eckstolonol	*Ecklonia cava*	Korea	Protect UVB-induced cell damage	[101]
Diphlorethohydroxycarmalol	*Ishige okamurae*	Korea	Inhibit MAPK; MMP-1; -2; -9 expression (↓) Pro-inflammatory mediators (↓) cellular damage	[114,115,116]
Fucodiphlorethol G	*Ecklonia cava*	Korea	(↓) DPPH, intracellular ROS; caspase-9 UVB absorption	[117,118]

Abbreviations: Nuclear factor kappa-light-chain-enhancer of activated B cells (NF-κB); Activator protein 1 (AP1); matrix metalloproteinase (MMP); Ultraviolet B (UVB); Reactive oxygen species (ROS); Bcl-2-associated *X* protein(Bax) [119], Nuclear factor erythroid 2–related factor 2 (Nrf2); Heme oxygenase-1 (HO-1); superoxide dismutase 1 (SOD-1); Glutathione (GSH); Mitogen activated protein kinases (MAPK); Down-regulated, decreased (↓); Up-regulated, increased (↑).

**Table 5:** Mycosporine like amino acid extracted from different seaweed species.

Species	Origin	PI	AS	SH	PR	Myc-gly	Usu+PI	PL	CL	Total	Ref
*Ahnfeltiopsis devoniensis* (mg/g)	Spain	NA	NA	0.55	NA	NA	NA	NA	NA	NA	[127]
*Curdiea racovitzae* (μg/mg)	Antarctic	111.49	36.51	2.17	NA	NA	NA	NA	NA	150.17	[27]
*Catenella repens* (mg/g)	France	NA	NA	NA	NA	NA	NA	NA	1.76	NA	[128]
*Catenella caespitosa* (mg/g)	Puerto Rico	NA	NA	NA	NA	NA	NA	NA	1.06	NA	[128]
*Gelidium corneum* (mg/g)	Spain	0.13	0.47	0.1	NA	NA	NA	NA	NA	NA	[127]
*Gracilariopsis longissima* (mg/g)	NA	NA	NA	NA	NA	NA	NA	NA	NA	1.6	[129]
*Gracilaria birdiae* (mg/100 g)	Brazil	14.67	NA	52.70	178.39	NA	NA	NA	NA	245.77	[130]
*Gracilaria caudate* (mg/100g)	Brazil	34.55	NA	32.20	48.15	NA	NA	NA	NA	114.90	[130]
*Gracilaria domingensis* (mg/g)	Brazil	10.41	1.25	7.56	28.82	NA	NA	1.54	NA	49.59	[130]
*Hydropuntia cornea* (mg/g)	NA	NA	NA	NA	NA	NA	NA	NA	NA	0.8	[129]
*Iridaea cordata* (μg/mg)	Antarctic	49.45	7.58	3.75	NA	NA	NA	NA	NA	60.78	[27]
*Palmaria palmata* (µmol/g)	Japan	2.964	0.078	0.155	1.900	0.276	0.276	NA	NA	5.372	[131]
*Palmaria palmata* (mg/g)	UK	9.94	0.08	0.63	0.56	NA	NA	0.11	NA	NA	[128]
*Porphyra rosengurttii* (mg/g)	Spain	0.17	0.14	0.38	3.84	NA	NA	NA	NA	NA	[127]

Abbreviations: Palythine (PI); Asterina-330 (AS); Shinorine (SH); Porphyra-334 (PR); Mycosporine-glycine (Myc-Gly); Usujirene (Usu); Palithynol (PL); Catenelline (CL); not available (NA).

**Table 6:** Fucoxanthin extraction from *Undaria pinnatifida* using different extraction techniques.

Extraction Method	Solvent Extraction	Solvent Extraction	SFE	SFE	UAE	MAE
Solvent	MeOH (1:50 *w/v*)	MeOH (1:50 *w/v*)	CO_2_ and EtOH (3%, *v/v*)	CO_2_	Deionized H_2_O (1:100 *w/v*)	EtOH (15:1 *w/v)*
Pretreatment	Wash, salted, boiled, blanched, cured	Avoid sunlight	Freeze dry	Milling and microwave assisted cell disruption	NA	NA
Extraction condition	1 h, RT	1 h, RT	50 bar, 200 °C, 1 h	40 bar, 400 °C, 3 h	800 W, 80% amplitude, 20 kHz, 30 °C, 3 h.	ratios, 60 °C, 10 min, 300 W
Yield	2.08 ± 0.04 mg/g	4.96 ± 0.4 mg/g	0.00753 μg/g	38.5 mg/g	0.031 mg/g	2.12 mg/100 g
Notes	Processed	Fresh	Pressure and temperature affect yield	MW pretreatment increased fucoxanthin yield	Sporophyll; small pilot scale	No effect on microwave power
Ref	[173]	[173]	[174]	[175]	[176]	[177]

Abbreviations: Microwave assisted extraction (MAE); Ultrasound assisted extraction (UAE); Supercritical fluid extraction (SFE); Methanol (MeOH); Ethanol (EtOH); Carbon dioxide (CO_2_;, Molecular weight (MW); Weight/volume (*w/v*), Volume/volume (*v*/*v*); Room Temperature (RT).

**Table 7:** Anti-aging and photoproctive ingredients from seaweeds available in the market.

Algae Species	Trade Name	Company	Active Ingredients	Anti-Photoaging	Ref
*Poprphyra umbilicalis*	Helionori^®^	Gelyma, French	MAAs	Photoprotective (UV-A) DNA protection Prevention of sunburn	[179]
*Poprphyra umbilicalis*	Helioguard365	Mibelle Biochemistry, Switzerland	Porphyra-334 and Shinorine	Photoprotective (UV-A)	[180]
*Poprphyra umbilicalis*	Algae gorria; Alga marris	Laboratoires de biarritz, French	NA	Photoprotective (UV-A)	[181]
*Undaria pinnatifida*	Fucorich	Marinova, Australia	Fucoidan	Anti-aging	[182]
*Fucus vesiculosus*	Maritech reverse	Marinova, Australia	Fucoidan	Anti-aging; antioxidant; anti-inflammation	[182]
*Fucus vesiculosus*	Maritech synergy	Marinova, Australia	Fucoidan and polyphenol complex	Anti-aging; antioxidant; anti-inflammation	[182]

Abbreviations: Mycosporine like amino acids (MAAs); Ultraviolet (UV).
